# Researches Tending to Prove the Non-Vascularity and the Peculiar Uniform Mode of Organization and Nutrition of Certain Animal Tissues, &c.

**Published:** 1842-04-01

**Authors:** 


RESEARCHES TENDING TO PROVE THE NON-VASCULARITY AND THE PECULIAR UNIFORM MODE OF ORGANIZATION AND NUTRITION OF CERTAIN ANIMAL TISSUES, VIZ. ARTICULAR CARTILAGE, AND THE CARTILAGE OF THE DIFFERENT CLASSES OF FIBRO-CARTILAGE; THE CORNEA, THE CRYSTALLINE LENS, AND THE VITREOUS HUMOUR; AND THE EPIDERMOID APPENDAGES. By *Joseph Toynbee*, Esq. Member of the Royal College of Surgeons in London, and late Assistant to the Conservators of the Museum of that Institution. (From the Phil. Trans. Part II. for 1841.) London, J. & E. Taylor, 1841.

THOSE who enjoy the pleasure of Mr. Toynbee's acquaintance are aware of his zeal and indefatigable assiduity in the prosecution of anatomical researches. He has brought to bear on them the practical experience derived from his studentship at the College of Surgeons, where he worked under the direction, and, we may reasonably suppose, imbibed the spirit, of the accomplished Conservators of the Hunterian Museum. From Mr. Toynbee therefore his friends expected much, and they will not, we think, be disappointed.

The title-page of the paper before us explains its author's object. We may therefore pass at once to its analysis.

Mr. Toynbee starts with the observation that it is now generally acknowledged that the process of nutrition in most animal tissues consists in changes undergone by the nutrient liquor sanguinis, which has exuded into them through the coats of the capillaries ramifying throughout them. The vessels themselves vary in number in different structures: in muscle, the capillaries are very numerous, and the spaces between them very small; whilst in tendon and ligament, on the other hand, the latter are comparatively large; but in all structures, whatever may be the degree of their vascularity, the tissue the furthest removed from the vessel is nourished equally well with that which is in immediate contact with it.

In all vascular structures, therefore, there is of necessity a considerable extent of tissue which is nourished without being in contact with bloodvessels, and the knowledge of this fact forms a necessary introduction to the study of the process of nutrition in those organs, into which, whilst in a healthy state, anatomists have never succeeded in tracing blood-vessels. The organized tissues, constituting such non-vascular organs, may be divided into three classes:

*The first*, comprehending articular cartilage, and the cartilage of the different classes of fibro-cartilage;

*The second*, the cornea, the crystalline lens, and the vitreous humour;

*The third*, the epidermoid appendages, viz. the epithelium, the epidermis, nails and claws, hoofs, hair and bristles, feathers, horn, and teeth.

Mr. Toynbee's endeavour is to prove that no vessel ever enters these tissues when they are perfectly developed, and in a healthy state, and to demonstrate the manner in which they are nourished.

Mr. Toynbee adopts the following line of argument.

In the first place, no anatomist has ever been able to trace vessels into these tissues in the adult healthy state; Secondly, he thinks that the due action of the organs into the composition of which these tissues enter is incompatible with vascularity; in other words, that the presence of blood-vessels within them is a sign of disease.

The first class, for instance, of the non-vascular tissues, comprising cartilage and fibro-cartilage, is subject, from its situation in the joints, &c., to repeated concussions, and to constant compression and attrition, which in these unyielding tissues would necessarily be destructive of the integrity of blood-vessels.

Those of the second class are required to be perfectly transparent for the due transmission of the rays of light, which would be impossible if the circulation of a coloured fluid were carried on throughout their substance.

The tissues of the third class are unceasingly exposed to friction, laceration and incision; and hence, of course, it is necessary that they should not be traversed by vessels.

Mr. Toynbee's injections have proved to him that the vessels which previous anatomists had traced no further than their circumference (supposing them to be continued into these tissues, either as serous vessels, or as red blood-vessels too minute for injection), actually terminate in veins, without the limits of these tissues. The terminations of these vessels in the immediate vicinity of the non-vascular tissues, present certain convolutions, dilatations, plexuses and other peculiarities, which differ in various parts, but in all instances enable a large quantity of blood to circulate slowly in the neighbourhood of these tissues, from which it may be inferred that they are subservient to the nutrition of the latter; and their existence certainly constitutes another argument against the presence of vessels within them.

All these non-vascular tissues contain corpuscles or cells, of which some of them are almost entirely made up; while in others, as the cornea, a few only are present.

Mr. Toynbee refers to the researches of Schwann, &c., on the cells of animal tissues. These cells are modifications of two principal forms, the one being either round or oval, the other compressed in the form of a scale; and their existence in one of these two forms has lately been detected in most organs of the body.

In a circular form they appear to be the principal components of bone, cartilage, muscular and nervous fibre, and of the parenchyma of glands; in the form of a flattened cell or scale, they constitute the epidermis and its appendages, the epithelium of mucous and serous membranes, and of the inner tunic of the vascular system.

Mr. Toynbee agrees with M. Schwann that these cells have vital actions, and, he thinks, not only during development, but afterwards. He ascribes to them the function of circulating, and of perhaps changing the nature of, the nutrient fluid which is brought to the circumference of the solid non-vascular tissues, and he believes them in some measure to compensate for the absence of the internal vascularity possessed by other structures. The only difference, in fact, which appears to him to exist between the mode of nutrition in the vascular and the non-vascular animal tissues is, that *the former* derive their nutrient fluid from the blood which circulates through the capillaries contained in their substance; and that *the latter* are penetrated by the nutrient fluid which exudes from the large vessels by which they are surrounded, and that its distribution through them is assisted by the vital properties of the corpuscles which they contain; in *both classes*, the particles of which the tissues are composed attract from this fluid the elements which nourish them.

### FIRST CLASS OF NON-VASCULAR ANIMAL TISSUES.

#### *Of Articular Cartilage and Fibro-Cartilage.*

These tissues, says Mr. Toynbee, are analogous to each other in their situation, structure, mode of nutrition and functions. Each of them forms a part of joints, and is subject, in the performance of its functions, to concussion and compression, and is composed of corpuscles or cells possessing similar characters. Although they are properly considered as non-vascular tissues, they appear to be pervaded by blood-vessels at an early period of their development, or perhaps it would be more correct to say, that as growth proceeds, the cartilage increases, so as to occupy the space which had previously been permeated by vessels. Mr. Toynbee thinks he has been able to demonstrate that vessels are never found within these cartilages when fully developed, but at that period vessels form convolutions in their immediate vicinity. These vessels are separated from articular cartilage at adult age by a layer of bone, and in fibro-cartilage, at the same period, they uniformly terminate within the boundary of its fibrous tissue. Over a certain portion of the free surface of both of these tissues blood-vessels extend, but they do not penetrate into their substance.

He thinks that his investigations lead to the certain conclusion, that articular cartilage in the adult state is principally nourished by fluid derived from the vessels of the cancelli of the bone to which it is attached, which exudes through the coats of those vessels, and makes its way into the substance of the cartilage through the intermediate lamella of bone. The cartilage of fibro-cartilage is nourished in like manner by liquor sanguinis, derived from vessels situated in the contiguous fibrous portion. The vessels ramifying in a certain extent of the free synovial surface of both these species of cartilage contribute doubtless to their nutrition, but not to near the same extent as do the vessels of the opposite side.

### 1. *Articular Cartilage.*

Mr. Toynbee observes that there is this difference in articular cartilage, with regard to its nutrition during and after its development; that in the former state there is no positive separation of it from the cartilage which is subsequently converted into bone, and in which its nourishing vessels are contained; whilst, in the latter state, these vessels are separated from it by an osseous lamella. The free surface of articular cartilage during, as well as after, its development, is covered by synovial membrane, to which it is attached by cellular tissue. So, it will be observed that Mr. Toynbee affirms what some have, we think, hypercritically denied—the continuity of synovial membrane over articular cartilage.

With respect to vascularity, the nutrition of articular cartilage during its development may be divided into two stages; viz. the early one, during which no vessels enter any of the structures of the joints; and the subsequent one, in which the cartilage of the bone on the one surface, and the synovial membrane on another, are supplied with blood-vessels. In adult age, after development is completed, the same vessels continue the process of nutrition; those of the bone being situated in the cancelli, as before described, whilst those of the synovial membrane are considerably diminished in size.

*First Stage of Development of Articular Cartilage.*—Mr. Toynbee has made some dissections of the fœtal calf with the view of illustrating this. The dissections themselves we cannot give, but must content ourselves with stating what Mr. Toynbee deems fair inferences from them.

*First*, That, during the most early periods of fœtal life, the growth of cartilage takes place, and that its component cells or corpuscles undergo certain progressive changes in their form and size, without the presence in its substance of any blood-vessels.

*Secondly*. That the vessels encircling the cartilage contribute to effect such changes in its corpuscles, and that the changes are facilitated by the softness of the substance of the cartilage.

*Thirdly*. That, at the more early period of fœtal development, the synovial surface of cartilage does not contain blood-vessels.

*Second Stage of Development of Articular Cartilage.*—As the cartilage forming the articular extremities of bones becomes harder during the process of development, vessels are gradually introduced upon its surface and into its substance. Mr. Toynbee treats of the latter first.

*Of the Blood-vessels in the Substance of the Epiphysal Cartilage.*—Mr. Toynbee observes that " the whole inferior extremity of the os femoris of a fœtus of about five months presents, except at its articular surface, numerous depressions of various depths. The deepest may be regarded as canals, some of which are single, others bifid; they terminate in blind sacs. The direction of some of these canals is towards the centre of the epiphysis, of others towards its point of attachment to the osseous shaft, and of others, those about to be described, towards the articular cartilage. Some of these canals are of a large size, and are frequently considerably dilated at their blind extremities. They do not penetrate into the substance of the articular cartilage. These canals are for the reception of branches of sanguiferous vessels. When the epiphysis is minutely injected, the depressions upon its surface will be found to contain congeries of convoluted blood-vessels, which are more drawn out the deeper the depression, until at length, in the interior of the canals and their divisions, single, and nearly straight vessels are found. These epiphysal vessels have a very peculiar disposition. They consist of an artery having a course more or less straight, which terminates in a dilatation, or in convoluted branches, from which the vein arises. From the fact of the presence of these vessels, which converge towards and form convolutions internal to the articular cartilage, it may be inferred that they supply the cells of the latter with a nutrient fluid. As the articular cartilage increases in thickness, and the ossific nucleus which is developed in the epiphysal cartilage becomes larger, these vessels gradually recede from between them, and they leave a considerable mass of non-vascular cartilage between the osseous nucleus and the synovial membrane; all of this appears to be articular cartilage, which is now nourished by the vessels in the interior of the nucleus. The supply of blood-vessels in the cancelli of the osseous nucleus, is remarkably abundant; they are large and are separated from the surrounding cartilage by an extremely delicate lamina of bone, which is principally made up of osseous cells. I am induced to believe that at this stage of development, as in adult age, the fluid passes from the bone into the cartilage and nourishes it."

*Of the Nutrient Vessels of Articular Cartilage during its Development, which are situated betwixt it and its Synovial Membrane.*—Mr. Toynbee commences by stating his belief in the extension of synovial membrane over the free surface of articular cartilage.

The arteries, he says, passing between the synovial membrane and the articular cartilage may be considered as the terminal branches of the articular vessels.

Before they reach the articular cartilage they are but laxly covered by the synovial membrane, but at the border of the cartilage they are firmly bound down to it by the very small quantity of dense cellular tissue existing between them. It is difficult to state generally at what period of fœtal existence the vessels, which have been spoken of in the first stage as forming convolutions around the joints, are prolonged upon its surface. Mr. Toynbee offers a detailed account of the prolongation of these vessels on the head of the os femoris from the point of insertion of the ligamentum teres, but we have not room for it.

*Various Characters of the Synovial Vessels.*—These vessels, remarks Mr. T., consist of arteries which take a direction towards the centre of the articular cartilage, and of veins which take a retrograde course. The arteries become continuous with the veins in the following ways; 1st, the artery becomes directly continuous with the vein, without undergoing any change in its size, forming with the latter a simple loop; 2ndly, numerous arteries terminate in a single vessel from which veins arise; 3rdly, the artery terminates in largely dilated vessels, from which the veins take their origin.

Mr. Toynbee adds:—the preceding account of the examination of the vessels of articulations at early periods, shews that a large quantity of blood-vessels exists both at the free and attached surface of articular cartilage during its development. The modes in which these vessels are disposed, the dilated, plexiform, and other characters which they present at the point of communication of the arterial with the venous system, are interesting features in the anatomy of the vascular system, and their presence here must be associated with the large quantity of fluid required for the nutrition of the articular cartilage during development, and which is eliminated from the blood whilst its course is retarded in these vessels.

*Adult Articular Cartilage.*

Mr. Toynbee has previously remarked that, at the attached surface of adult articular cartilage, viz. at the part where it joins the osseous lamella, it presents numerous fine canals, which can be seen only with the higher magnifying powers. These canals are irregular in their distribution; some are merely dilated cavities; frequently several of these cavities are elongated, and arranged serially, running from the attached towards the free surface of the cartilage. At the free or synovial surface, these canals do not exist; the cells of the texture at this part being elongated and flattened, and having their long diameters parallel to the free surface. These canals contain a transparent fluid, which is seen to ooze from them after a section.

It is most probable that the uninjected vessels observed in sections of cartilage by Meckel, Bichat, and others, were these canals and sinuses.

Into the substance of healthy articular cartilages Mr. T. has never been able to trace blood-vessels, and he believes that they do not possess any. Mr. Toynbee quotes the contradictory opinions of the most eminent anatomists and physiologists upon the subject. He then continues:—

In adult life, when the epiphysal cartilage has been ossified, the cancelli of the latter are separated from the articular cartilage by a layer of bone, to which may be given the name of the articular lamella. The nature of this lamella is worthy of particular attention. It is composed of two sets of osseous layers; the one, dense and thick, is continuous with the vertical fibres of the cancelli; the other, delicate and thin, principally composed of osseous corpuscles, is situated at right angles to the latter, and fills up the interspaces of the vertical fibres. Mr. Toynbee asks if this lamella is complete, and replies that he never could discover its orifices, nor force mercury through it.

"If the lamella be rendered transparent by varnish, or its earthy particles are removed by acid, numerous vessels will be perceived through it in the interior of the cancelli, which in some measure permeate the osseous articular lamella, rendering it transparent, and their contents may then be seen and examined. To facilitate this examination, I have in some instances resorted to the application of varnish, and in others have removed the earthy particles from the bone by the aid of acid. Through the articular lamella numerous vessels of considerable size will be distinctly recognized in the interior of the cancelli. These vessels enter the substance of the bone by the large foramina which are seen at its non-articular surfaces, and they converge towards the articular lamella. With the inner surface of this lamella, they not unfrequently appear to be in contact; and either in contact with it, or near to it, these vessels form dilatations and convolutions, and then take a retrograde course and become continuous with the venous system. Mr. Toynbee adds:—

"I believe that the large vessels which I have already described as forming convolutions and dilatations at the inner surface of the articular lamella, have the function of supplying the articular cartilage with a nutrient fluid, and that they do so without entering into its substance. It is necessary that the nutrient fluid brought to the inner surface of this lamella should penetrate its substance. It is most probable that it traverses only the thin layer of the lamella, and not the vertical portions. This thin layer has already been stated to be almost entirely composed of osseous corpuscles, which without doubt assist to convey the fluid from the cancelli into the cartilage.

It appears to me, that not only those vessels which are in immediate contiguity with the articular lamella have the function of nourishing the articular cartilage, but that the large and very numerous vessels which ramify through the substance of the cancellous extremities of bones, and which enter them by large orifices at their non-articular circumference, eliminate into the cancelli a nutrient fluid, which passes through the articular lamella and nourishes the cartilage.

That the nutrition of articular cartilage is actually effected by vessels in the cancelli, may be inferred from their dilatations and convolutions in its vicinity, and from the absence of any other means, as shown by my injections." 172.

He refers to a preparation of Mr. Swan's, and to some unpublished observations of his own on the morbid conditions of cartilage, in which blood-vessels *are* prolonged into its substance, in corroboration of his views.

Mr. Toynbee next proceeds to shew that *no vessels enter articular cartilage at its free synovial surface.* Mr. Toynbee thus speaks of the—

*Vessels of the Synovial Membrane which cover the Articular Cartilage, and of the Nutrition by them of the latter.*

During fœtal and infantile life, he says, previous to the period when the articular cartilages are subject to pressure, the synovial vessels extend over certain portions of them, from which in childhood and during adult age, owing to the functions of the joints, they are necessarily absent. At the period when the child begins to use the various joints, and subjects them to pressure, these vessels recede; and in adult life they are only found on that margin of articular cartilage which is exempt from the influence of external forces. The arteries which pass between the articular cartilage and the synovial membrane, like those of the fœtus, may be considered as the termination of the articular arteries. At the point where the reflexed becomes continuous with the articular synovial membrane, it contains large vessels subjacent to it, which are numerous and plexiform. Immediately, however, that they enter the cellular web, between the articular cartilage and synovial membrane, they become enlarged and straight, and pass to a greater or less distance over the border of the articular cartilage, forming loops frequently with considerable dilatations, and becoming finally continuous with the veins. The free surface of adult articular cartilage appears to be nourished by the liquor sanguinis, which exudes from these looped and dilated vessels.

Mr. Toynbee thus sums up the leading inferences from his researches on *Articular Cartilages.*

1. Epiphysal and articular cartilage are developed and nourished in the early periods of fœtal existence, without the presence of blood-vessels in the substance of the former, or on the surface of the latter.

2. At subsequent periods, canals are formed in the epiphysal cartilage, vessels are prolonged into them, which converge towards the articular cartilage; and also vessels extend over a considerable portion of the free surface of articular cartilage.

3. At later periods, the epiphysal cartilage ossifies, and for a considerable time vessels are placed between the ossified nucleus and the articular cartilage.

4. As age advances, the osseous nucleus increases in size, the blood-vessels disappear from the cartilage which remains unossified, but the nucleus contains large and numerous blood-vessels.

5. Corresponding with the changes just noticed, is the recession of the blood-vessels from the whole of that surface of the articular cartilage which is subject to compression.

6. In adult life, the articular cartilage contains no blood-vessels; but in the cancelli of the bone at its attached surface, are numerous large vessels, from which the cartilage is separated by a delicate lamella of bone; the circumference of its free surface presents numerous dilated blood-vessels.

7. Articular cartilage during the whole of life gradually becomes thinner, by being converted into bone.

### 2. *Fibro Cartilage.*

After noticing the contradictory opinions of anatomists, Mr. Toynbee states, that he has always found fibro-cartilage composed of minute fibres, and of the corpuscles characteristic of cartilage. He recognises the division of the fibro-cartilages into two great classes—the first deprived of synovial membrane, as the intervertebral and symphytic fibro-cartilages—the second having two surfaces covered with synovial membrane, as the inter-articular. In both classes the fibrous portion of the tissue is most abundant towards the circumference, and in this portion only are blood-vessels found. In some instances, the centre of the fibro-cartilage is entirely composed of cartilaginous corpuscles. The cartilaginous portion is comparatively more abundant in young than in adult subjects, and in the latter it diminishes as age advances. This diminution of the cartilaginous portion is, doubtless, to be attributed to the gradual conversion of the cartilaginous corpuscles into fibres.

Mr. Toynbee, gives an account of the intervertebral substance of the mackerel, the cod, the porpoise, and the dog. In all, the above statement is borne out. But we pass to the intervertebral substance of man at the various periods of existence.

*a*. In the human fœtus of the *third month* the intervertebral substance of the second and third lumbar vertebræ is firm and white at its circumference, soft and of a leaden hue towards its centre. The central part is composed of numerous cells or corpuscles, some being round, others stellated. It also presents large masses of other cells aggregated together, and somewhat of a darker colour. Nearer to the circumference of the intervertebral substance the cells are arranged in distinct lines, and at its circumference they are elongated at each extremity, and so attenuated as to assume the appearance of fibres.

*b.* In a human fœtus of *seven months* the intervertebral substance is composed of the external fibrous and the central cartilaginous portions. The central portion is almost entirely composed of distinct round cells, which are found, on being traced towards the fibrous part, to become more elongated at their extremities, and to form, as it were, series of fibres. The fibrous portion is made up of circular layers of fibres, between which cells are interspersed.

*c.* In a human fœtus of *nine months* the central part of the intervertebral substance is soft, and is composed of cells, and of an intercellular substance. The external fibrous portion is distinct, and is interspersed with cells.

*d.* From the period of birth to adult age, the change undergone by the intervertebral subtance, consists in the gradual encroachment of the fibrous portion upon the cartilaginous.

*e. In adult age* the central portion still continues to present corpuscles, although not in so great a number. They are always found interspersed through a gelatinous mass. The external fibrous portion also presents corpuscles between its circles of fibrous tissue.

*f. In old age* the corpuscles of the intervertebral substance are less numerous than in the abult. The fibrous tissue is also more dense and unyielding.

The symphytic fibro-cartilages present very similar conditions and changes.

### *Second Class of Fibro-Cartilages.*

They, like the fibro-cartilages of symphyses, consist of an external fibrous and of a central cartilaginous portion. At the early periods of their development, the cartilaginous is more abundant than the fibrous portion, and it is almost entirely composed of corpuscles. As age advances, the fibrous portion increases in quantity; and towards the later periods of life, the corpuscles of the cartilaginous division are mixed with fibrous tissue.

*Vessels of Fibro-Cartilage.*—From injections of the intervertebral substance of man and animals, Mr. Toynbee has found that the external more fibrous portion is pierced by arteries of considerable size; these are guarded from compression by the dense nature of the fibrous tissue through which they pass. They course towards the central cartilaginous portion, into which, however, they do not penetrate, but in its confines they form large convoluted dilatations, from which the recurrent vein arises. The extreme edge of these vascular convolutions presents a line which may be considered as the boundary between the fibrous and cartilaginous portions of the intervertebral fibro-cartilage.

Very analogous appearances are observed in the fibro-cartilages of the symphyses.

*Inter-articular Fibro-Cartilage.*—"The central part of the inter-articular fibro-cartilages in the injected specimens that I have examined from the human fœtus as early as the third or fourth month, does not contain any vessels. I possess, however, the inter-articular cartilage of the temporo-maxillary articulation of a fœtal calf, which is pervaded by blood-vessels throughout its entire substance; a disposition which may take place in all fibro-cartilages at very early periods of their development. Subsequent to these very early periods, however, the central portion, which, like articular cartilage, is subject to concussion and compression, does not contain any blood-vessels." 178.

Of the inter-articular fibro-cartilages generally, it may be observed that arteries of some size pierce, and are arranged in different ways in the external fibrous portions, and converge to the cartilaginous portion, into which they do not penetrate.

Vessels extend to a short distance on the surface of fibro-cartilages, beneath the synovial membrane, but they are arrested at the part where these structures are subject to pressure, and at this margin they form dilatations similar to the synovial vessels which cover the border of articular cartilages.

### Second Class of Non-Vascular Animal Tissues.

This class comprises the *Cornea, Crystalline Lens,* and *Vitreous Humour.* These may be considered as constituting a class of non-vascular organized tissues, inasmuch as each of them is transparent, each forms a part of the eye-ball, and performs a similar function, viz. transmitting the rays of light to the retina. These three structures are nourished by the penetration into them of a nutrient fluid, which is derived from the numerous blood-vessels which encircle them; although each of them contains corpuscles, they differ from each other in their structure as well as in their relations with the vascular system.

### *Of the Cornea.*

Mr. Toynbee first notices, to dispute, the statement of Müller, that the cornea has no corpuscles. Some of them are rounded, others are oval, and have fine branches radiating from them, similar to the osseous and pigment corpuscles. These cells are surrounded by the bright fibres of which Müller has spoken; these fibres, which compose the larger portion of the cornea, are laxly connected together, so as to have some analogy with cellular tissue; the substance of the cornea being of a loose texture, and easily pervaded by fluids. Mr. T. thinks that this easy penetration prevents the necessity for as many corpuscles in this as in the denser tissues.

Mr. Toynbee next describes the vessels which nourish the cornea, previously quoting the opinions of Boyer, Cruveilhier, Jacob, Lawrence, Romer, Tyrrell, and Wardrop. Mr. Toynbee then goes on to state:—

In an eye which is injected with tolerable success, the white sclerotic membrane will be observed to be traversed by two sets of vessels. One of these consists of small and numerous branches, which have a straight direction towards the circumference of the cornea. These are the ultimate branches of the sclerotic arteries which course towards the cornea. The other set of vessels is composed of the large and tortuous trunks which are seen with ease by the naked eye; these are the sclerotic veins, which take a retrograde course to the arteries just alluded to, and become gradually larger as they get more remote from the cornea; these sclerotic veins return the blood devoted to the nutrition of the cornea.

Upon examining *the arteries* with a magnifying glass, they will be found, at the circumference of the cornea, to terminate in two sets of vessels; of these, one is superficial, and consists of delicate branches which pass inwards over the surface of the cornea, between it and its mucous covering, and are analogous to the vessels of joints which pass between the articular cartilage and the synovial membrane. The other set of vessels in which the sclerotic arteries terminate, are much larger than those just noticed, and are more like the continuation of their trunks; these, at the circumference of the cornea, pass into the substance of the sclerotic, where they come in contact with the attached margin of the cornea.

The *superficial or conjunctival* arteries, upon arising from the sclerotic, take a course parallel with the circumference of the cornea, and are sometimes so long as to receive the name of its circular vessels: from these, branches are given off which pass in a direction towards the centre of the cornea. These, after division and sub-division, form a minute plexus on the border of the latter, and they terminate on its surface from one-eighth to half of a line from its circumference, by becoming continuous with the venous system, in several modes.

The small *veins* which arise from the arteries take an opposite course to the latter, and upon reaching the margin of the sclerotic, they empty themselves into the large tortuous veins, which have been noticed above, just as the latter are emerging from the substance of the sclerotic, where it is attached to the cornea.

The *deep or sclerotic arteries* of the cornea are those from which this structure derives its chief nutrition. They are large enough to be considered the continuation of the trunks of the sclerotic arteries; they pass without much diminution of their size towards the point where the sclerotic membrane is continuous with the cornea. At the margin of the latter they suddenly stop and turn back, forming loops; sometimes with, and at other times without dilatations, they become continuous with the veins. These veins emerge from the substance of the sclerotic, close to the margin of the cornea, at which point they receive the conjunctival veins: and they take a backward course, and form those tortuous veins which have already been noticed.

But, in disease, the marginal vessels of the cornea are prolonged through its substance, while those which, when healthy, extend over the surface of the cornea from the one-eighth to half of a line, in disease form a band of considerable extent.

*The Crystalline Lens.*

M. Schwann thinks, that the lens is at first composed of cells, which are afterwards converted into fibres. Mr. Toynbee has not only found cells interspersed among its fibres, but has frequently seen the fibres themselves composing the external part of the lens, made up of these cells; and in other instances they occupy the margin of the fibres only.

Speaking of the *blood-vessels* of the lens, Mr. Toynbee says—"I have not only been unable to trace vessels into the anterior capsule, but I hope to prove that in the healthy state no vessels do enter it. The posterior capsule of the lens is, however, injected with facility, and contains large and numerous ramifications of blood-vessels; I ascribe to them the function of supplying the crystalline lens with a nutrient fluid. These vessels arise from the arteria centralis retinæ; the latter, having traversed the centre of the vitreous humour, expands upon the capsule, and forms the ramifications just noticed. Now in some injections which I have made of the eyes of a human fœtus, of the sixth or seventh month, these vessels were not confined to the posterior surface of the capsule; they pass round its border and extend upon its anterior face to the extent of one quarter of a line. I have not been able to make a perfect injection of the vessels of the capsule of the lens in ages antecedent to the fifth or sixth month of the fœtal life, and therefore am unable to say whether, in the very early periods of development, the anterior capsule, like the membrana pupillaris, is entirely traversed by vessels; the crystalline lens would, under such circumstances, be completely surrounded by blood-vessels.

The branches of the arteria centralis retinæ in the early periods of life, as noticed above, extend upon the anterior surface of the capsule. Immediately they reach the latter they become straight, run parallel with each other, and are directed towards the centre of the anterior surface for the distance of a quarter of a line, when they stop in their course, and form looped dilatations, which give origin to small veins. It is most probable that these vessels recede at subsequent periods of development, so as to leave the whole of the anterior surface of the capsule capable of being permeated by the rays of light. These vessels, in a diseased state, are sometimes prolonged into the whole of the anterior capsule, (or to speak with more propriety, the anterior half of the capsule,) where, in morbid specimens, they have been injected by Schroeder Van der Kolk. The capsule of the lens is thus pervaded by large and numerous ramifications of blood-vessels, which I believe pour out upon its inner or lenticular surface a nutrient fluid; this *fluid will immediately come in contact with* the mass of delicate cells described by Schwann as situated between the lens and the capsule." Mr. Toynbee supposes that the nutrition of the lens is effected by these cells receiving the elements of the blood, and conducting them to the lens, through which they are diffused.

*Vitreous Body or Humour.*

The membrane of the vitreous humour is very delicate, and interspersed with corpuscles.

The arteria centralis retinæ passes through the vitreous body, but Mr. Dalrymple alone has succeeded in injecting from it the periphery of the hyaloid membrane.

Mr. Toynbee is inclined to think that the ciliary processes have the function of nourishing the vitreous body. "I have succeeded," he says, "in making very minute injections of the vessels of the ciliary processes, the disposition of which is remarkably beautiful; they have been most accurately delineated and described by Zinn. I have particularly to allude to the immense quantity of blood that their large size allows to be continually circulating through them, and to their plexiform character, which is productive of a slow circulation of the fluid they carry. At the free border of each ciliary process is a large single vessel, which is received into the base of the sulcus of the vitreous humour, and is in immediate contact with it. When it is remembered that the investing membrane of the vitreous humour is very delicate, and that it is made up of cells or corpuscles, and that it has in immediate contact with its free surface these large and numerous vessels, they may perhaps with great reason be considered as the organs of nutrition of the vitreous humour, by eliminating a nutrient fluid which penetrates into, and is diffused through, the substance of the latter."

### THIRD CLASS OF NON-VASCULAR ANIMAL TISSUES.

### *Of the Epithelium, the Epidermis, Nails and Claws, Hoofs of various kinds, Hair and Bristles, Feathers, Horn and Teeth.*

All these are extra-vascular tissues, developed on the surface of the chorion. Each of them is in contact, at its attached surface, with numerous and large branches of the vascular system, and, with the exception of the teeth, each is almost entirely composed of corpuscles or cells, which are of a somewhat circular form, where they are near to the vascular chorion, and are compressed and flattened where they are further removed from it. These tissues grow, by the addition to them, at their point of attachment with the chorion, of new cells, and from the increase in size of those already developed.

The *Epithelium* is composed of corpuscles which are round where they are in contact with the vascular chorion, and of others which are flat and in the state of scales situated at its free surface. Immediately subjacent to the epithelium the chorion presents ramifications of blood-vessels which nourish it, and which have different characters in different portions of the mucous membrane, according to the various functions which they have to perform.

The chorion of the *integuments* of the human subject, where it is subjacent to the thin and delicate cuticle, presents a minute and intricate network of blood-vessels. The arteries divide, subdivide, and form a network, from which the veins arise. The vessels of the chorion, however, are differently arranged, where it is covered by thick and dense cuticle. Thus, it is well known, that at the palms of the hands, the anterior surface of the extremities of the fingers, the posterior part of the heel and at the sole of the foot, the thick *epidermis* forming corns, &c., the arteries of the chorion are observed to terminate in numerous dilated loops, a disposition which bears a close analogy to certain synovial vessels. The vessels of the chorion not only secrete the perspiration, but also form the walls of the sweat-ducts.

The *Nails*, "where in cohesion with the vascular chorion, are more or less soft; and when portions of them at this part are examined by the microscope, they are seen to consist of corpuscles somewhat compressed, and connected together by a gelatinous substance. The harder part of the nail consists of compressed and transparent corpuscles.

Although the nails do not contain any blood-vessels, they change their colour and become friable under certain conditions of the circulation, thus showing that their component cells have the power of circulating through them the fluid which is brought to them by the blood-vessels, at their attached surface.

The nails are in contact with the vascular chorion at two points; their attached margin is inserted into a groove of the chorion, and the attached surface lies upon that portion which covers the dorsum of the terminal phalanges.

The vessels which have the office of supplying the nail with a nutrient fluid are large and numerous. The arteries take their origin from the digital trunks, and they converge towards the dorsal surface of the terminal phalanx, on which they ramify, and where they may be considered to terminate in two sets of vessels; one of which is devoted to the supply of the ungual groove, the other to the ungual surface of the phalanx. The ungual groove is very vascular; it presents the ramifications of arteries, which, after division and subdivision, form large plexuses in the margin of that part of the groove which overlaps the nails, and the arteries terminate in loops of considerable size. The vessels of the ungual surface are of a considerable size; they form large convolutions; these give off branches which, with others from the interior and lateral part of the phalanx, form an intricate capillary network, which is in immediate opposition with the attached surface of the nail."

*The Claws* and *Horns* of *Quadrupeds* and the *Claws* and *Beaks* of *Birds* are in contact, at their attached surface, with large vessels. In some instances the bone upon which they rest is perforated by foramina, the chorion subjacent to them, and between them and the bone, is very attenuated, and they appear to be nourished also by the vessels contained in the bone itself.

*Hoofs* seem condensations of cuticle. In adult age that part of the hoof which is in connection with the chorion retains its analogy with the cuticle, but at its free surface it becomes hard and somewhat friable. It appears to Mr. Toynbee that the use of the elongated tubes containing white soft matter, and which pervade the substance of the hoof of the horse, is to convey through its substance the fluid secreted at its attached surface. The chorion subjacent to the hoofs of animals presents two different characters, the one being that of numerous fine villosities, the other of compressed lamellæ; these are received into depressions on the surface of the hoof, and are composed of vessels which terminate in loops, possessing frequently considerable dilatations.

*Hair* and *Bristles*, of various kinds, in the neighbourhood of the vascular chorion, are composed of roundish corpuscles loosely connected; more remote from the chorion, their substance is harder; the corpuscles are flattened, and they appear to possess the characters of horn. The chorion, with which the hair is connected, consists of a papilla, which is inserted into the interior of the base of the hair, and of a sheath or capsule which surrounds it; both the papilla and capsule are supplied with large and numerous blood-vessels, which form loops and dilatations.

*Feathers* near to the vascular chorion consist of corpuscles more or less compressed; further removed from the chorion they are highly compressed, and present a similarity to the hair. The chorion to which the feathers are attached presents a pulp and capsule, which have a disposition of vessels analogous to those of the hair.

The *Teeth* are now considered to be permeated by an infinity of fine tubes, which are supposed to have the function of conducting from the surface of the vascular pulp, a nutrient fluid, which is distributed over the substance of the tooth. In this way may be explained the manner in which the teeth change their colour during diseases, being impregnated by the various fluids circulating in the system.*

Mr. Toynbee thus concludes his exceedingly interesting and valuable paper.

"I think it is established, as a general law in Animal Physiology, that certain tissues are capable of being nourished without the presence of blood-vessels within them: it has been shown that all these tissues are surrounded by large blood-vessels, which appear to have no other function than of supplying to them a nutrient fluid; and the way in which this nutrient fluid is conveyed into the substance of these tissues has been also pointed out.

The analogy between the extra-vascular animal and the vegetable tissues is manifest.

The application of the above-named law to the study of *Surgery*, in reference to the causes of the prolongation of vessels into the extra-vascular tissues, and to the measures to be adopted for the prevention and cure of those diseases which are dependent thereon; and to *Pathology*, in the investigation of the nature of morbid structures, particularly of those classes which contain no vessels,—will, I feel certain, be productive of interest and great advantage." 189.

---

### MORAL REVULSION IN THE TREATMENT OF INSANITY. By *F. Leuret*, &c. &c. Paris. 1841. (Preceded by the Report made to the Royal Academy of Medicine by MM. Louis, Pariset and Double. The Report was read at the Sitting of June 1st, 1841.)

M. LEURET's work consists, on the one hand, of some general remarks on the moral treatment of insanity, especially on the value and importance of moral *diversions* in combating mental alienation. It presents, on the

---

* "Recent investigators have thrown so much light upon the structure and the mode of growth of the *Epidermoid Class* of non-vascular tissues, that (as is apparent) I have added but few new facts in this department of my researches."

